# Continuous cropping changes the absorption and accumulation of elements in *Casuarina equisetifolia*, thus affecting its physiological properties

**DOI:** 10.1515/biol-2025-1283

**Published:** 2026-03-02

**Authors:** Hezhi Lin, Yuhua Wang, Miao Jia, Miaoen Qiu, Yulin Wang, Haibin Wang, Zeyan Wu

**Affiliations:** College of Life Sciences, Fujian Agriculture and Forestry University, Fuzhou 350002, China; People’s Government of FuqingCity, Fuzhou 350002, China; College of Life Sciences, LongyanUniversity, Longyan 364012, China

**Keywords:** *C. equisetifolia*, continuous planting obstacle, multi-element, root system, photosynthesis

## Abstract

Continuous monoculture affects elemental uptake in *Casuarina equisetifolia* (*C. equisetifolia*). In this study, *C. equisetifolia* with different numbers of continuous plantings was used to determine multi-element contents in rhizosphere soil, roots, and leaves using inductively coupled plasma-mass spectrometry (ICP-MS), and to assess the physiological indices of the roots and leaves. The effects of continuous planting on the uptake and transport of elements, and physiological characteristics of *C. equisetifolia* were also analyzed. The results showed that continuous planting of *C. equisetifolia* significantly reduced the enrichment capacity of its roots and leaves for certain elements. Specifically, continuous planting reduced Na and Ni enrichment in roots and N, K, Mg, Lu, Rb, and Zn enrichment in leaves. In terms of elemental transport, continuous planting resulted in a significant decrease in the transport of Mo, N, Nb, and Sr by *C. equisetifolia*. Physiological indice results showed that root activity, root cation exchange capacity, chlorophyll content, and net photosynthetic rate in *C. equisetifolia* tended to decrease significantly with an increase in the number of continuous plantings. In conclusion, continuous planting reduced the uptake, enrichment, and transport of beneficial elements in *C. equisetifolia*, which in turn inhibited root growth, decreased photosynthetic capacity, and ultimately stunted plant growth. This study provides an important reference for planting management and elemental regulation in continuous planting systems of *C. equisetifolia*.

## 1 Introduction

Soil is rich in a large number of mineral elements and serves as an important medium for plant cultivation. During the growth process, plants absorb these elements from the soil through their root system and transport them to different tissues, which in turn promotes plant growth [1]. The uptake of elements by different plants is specific: on the one hand, plants selectively absorb and concentrate certain elements due to their preference for them; on the other hand, environmental factors can alter a plant’s ability to absorb and concentrate different elements, thus affecting its growth [2], [3], [4]. The purpose of plant absorption of elements is to meet growth requirements. However, not all elements benefit plant growth – an increase in the enrichment and transport of beneficial elements promotes growth, while the opposite occurs with harmful elements. Especially under environmental stress, the absorption and transport of beneficial elements decreases, while the enrichment of harmful elements increases, inhibiting plant growth [5], 6]. Understanding the effects of environmental factors on the uptake and transport of elements in plants is therefore crucial for effective plant cultivation and management.

Continuous planting refers to repeated monoculture of the same plant in the same soil. Long-term continuous planting is likely to lead to changes in the physicochemical characteristics of the soil, which in turn affect plant growth, a phenomenon commonly known as the “continuous planting obstacle” [7], 8]. *Casuarina equisetifolia* belongs to the Casuarinaceae family of evergreen trees and has significant economic value. It is widely planted in coastal areas of Fujian, Guangdong, Hainan, and other provinces of China due to its well-developed root system and strong resistance. It is primarily used for soil and water conservation and tidal erosion prevention in coastal areas [9]. However, the soil in coastal areas is relatively unique, mainly sandy, and there are fewer suitable tree species with both economic benefits and soil and water conservation functions. As a result, *C. equisetifolia* is often replanted *in situ* after harvesting [10]. As the frequency of *in situ* replanting of *C. equisetifolia* increases, the phenomenon of “continuous planting obstacle” arises, which is reflected in a decrease in tree height by 23.7 %, diameter at breast height by 24.4 %, and volume by 29.0 % [11]. It is evident that continuous planting significantly impacts the growth of *C. equisetifolia*, limiting the sustainable development of its protected forest resources.

The “continuous planting obstacle” can alter the effectiveness of elements in the soil, which in turn affects the uptake and utilization of these elements by plants. It has been reported that after long-term continuous planting of cucumber, the content of effective elements in the soil decreases, resulting to an imbalance in soil nutrients, a reduction in cucumber’s ability to absorb and utilize nutrients, and a decrease in yield [12]. Continuous planting also reduces the uptake and transport of sulfur (S) by the soybean root system, which results in reduced yield [13]. In tobacco, continuous planting mainly reduces the content of boron (B), potassium (K), magnesium (Mg), and manganese (Mn) in the leaves, leading to an increase in the frequency of disease and inhibiting growth [14]. These examples show that continuous planting affects the uptake of elements by plants, which in turn affects their growth. In addition, some studies have found that the impact of continuous planting on plant element uptake can be mitigated by regulating soil elements. Lyu et al. [15] found that continuous planting of cucumbers primarily inhibited the uptake of essential macroelements (N, P, K, Ca, Mg) and trace elements (Fe, Mn, Zn). However, the exogenous addition of silicon (Si) can improve cucumber’s ability to absorb elements, promote root growth, and increase biomass. Han et al. [16] conducted a 3-year study on the addition of different elements to continuously planted soybean soils and found that the addition of nitrogen (*N*) increased soybean yield by 97.7 %, phosphorus (P) by 89.0 %, potassium (K) by 26.0 %, and trace elements such as zinc (Zn), manganese (Mn), boron (B), and magnesium (Mg) by 16.9 %, 13.2 %, 1.4 %, and 14.6 %, respectively. These findings highlight that continuous planting affects the element absorption of different plants, and adjusting soil elements can improve plants’ ability to absorb and utilize these elements, thus promoting growth. The key to soil element regulation is to clarify the effect patterns of element uptake and identify the main elements involved in continuous planting. Therefore, understanding the elemental enrichment and transport capacity of different plant tissues in continuous planting systems is crucial for soil element regulation and the effective cultivation management of continuous planting. However, little research has been conducted on how continuous planting leads to changes in elemental uptake by *C. equisetifolia*, which in turn affects its growth. Accordingly, this study was designed to analyze the effects of continuous planting on the uptake and transport of elements and physiological characteristics of *C. equisetifolia*. Using ICP-MS, we determined the multi-element content of the rhizosphere soil, roots, and leaves, as well as the physiological indices of roots and leaves at different stages of continuous planting, to clarify the effect of continuous planting on the uptake and transport of key elements and the growth of *C. equisetifolia*, with the aim of providing a basis for the cultivation and management of *C. equisetifolia*.

## 2 Materials and methods

### 2.1 Field experiment and sample collection

The experimental site of this study was located in a state-owned protective forest farm in Chihu Township, Hui’an County, Fujian Province, China (118°55′ E, 24°35′ *N*). The area is predominantly planted with *C. equisetifolia*, with a forest stand density of approximately 950 plants/ha^2^, a soil texture of wind-deposited yellow sand, a mean annual temperature of 19.8 °C, and an annual rainfall of 1,029 mm. The total area of the farm is about 433 ha^2^. The forest farm was first planted with *C. equisetifolia* in 1987, and a portion of the forest was harvested and replanted with *C. equisetifolia in situ* in 2011. In March 2018, three different plots were selected for replanting *C. equisetifolia* (height 0.8 m, diameter at breast height 0.9 cm) at the forest site: one plot that had not previously been planted with *C. equisetifolia*, one plot planted with a single continuous planting of *C. equisetifolia*, and one plot planted with two continuous plantings of *C. equisetifolia*. The total area of each plot was approximately 2,700 m^2^, with three independent replicates, each of which had an area of 900 m^2^ (30 m × 30 m). The planting density of *C. equisetifolia* was 950 plants/ha^2^, with 85 plants planted in each replicate. Thus, three experimental plots of *C. equisetifolia* with varying numbers of continuous plantings were established: the first planted *C. equisetifolia* plot (M1), the second continuously planted *C. equisetifolia* plot (M2), and the third continuously planted *C. equisetifolia* plot (M3). Management practices during the cultivation of *C. equisetifolia* were standardized according to the “Technical Regulation on Cultivation of Casuarina Seedlings and Trees” (LY/T 3092-2019) issued by the National Forestry and Grassland Administration of the People’s Republic of China [17].

In March 2022, rhizosphere soils from *C. equisetifolia* plots with different numbers of continuous plantings were collected for ICP-MS determination of multi-element content. Simultaneously, the net photosynthetic rate of *C. equisetifolia* leaves was measured. Roots and leaves of *C. equisetifolia* were also collected to determine physiological indices such as root activity, root cation exchange capacity, and leaf chlorophyll content. The rhizosphere soil and roots of *C. equisetifolia* was sampled using the “S” sampling method, in which 15 plants from each replicating plot were randomly selected. The deciduous layer was removed, and the upper soil was shoveled to a depth of about 30 cm. Fine roots were cut, and soil adhering to the roots was collected using a small brush in a self-sealing bag and thoroughly mixed, yielding approximately 400 g of rhizosphere soil per replicate. In addition, fine roots about 4 cm in length were collected, with about 15 g per replicate. Three independent replicates were performed for each experimental treatment. The collected rhizosphere soil and roots were placed in iceboxes and transported to the laboratory for immediate analysis. For leaf sampling, five randomly selected mature leaves from each replicate plot were harvested using high pruning shears, then mixed into one sample (approximately 300 g). Three independent replicates were used for each experimental treatment.

### 2.2 Determination of physiological indices

Physiological indices of *C. equisetifolia* were determined, including root activity, root cation exchange capacity, chlorophyll content, and net photosynthetic rate. The net photosynthetic rate was measured using the LI-6400XT Portable Photosynthesis System (Li-Cor, Lincoln, NE, USA) between 9 a.m. and 11 a.m. on a sunny day. The measurements were taken with a photon flux density of 1,500 μmol/m^2^·s, an ambient CO_2_ concentration of 380 ppm, and leaf temperatures maintained at 25–26 °C, with a vapor pressure deficit (VPD) of less than 1 kPa. Chlorophyll content was extracted using acetone and determined by UV spectrophotometry [18]. Root activity was measured using a plant root activity assay kit (Naphthalamine microplate method) provided by Beijing Leagene Biotechnology Co., Ltd [19]. Root cation exchange capacity was determined by KCl elution and KOH titration methods [20].

### 2.3 Element determination

#### 2.3.1 Sample preparation and element determination

The collected fresh roots and leaves of *C. equisetifolia* were rinsed with deionized water to remove adhering dust and impurities, then placed in an oven set to 80 °C until dried to a constant weight. The samples were then ground and passed through a 75 μm nylon mesh for subsequent sample digestion and elemental determination. The collected soil samples were air-dried at room temperature, ground, and passed through a 75 μm nylon mesh for further digestion and elemental analysis.

The same digestion method was applied to rhizosphere soil, roots, and leaves of *C. equisetifolia*. Briefly, 0.5 g of the sample was weighed, 5 mL of HNO_3_ was added, and the sample was digested at 185 °C for 4 h. After digestion, the acid was drained for 1 h. The digest was then transferred to a 50 mL volumetric flask and diluted with deionized water to the mark for ICP-MS elemental content determination. Three independent replicates were performed for each sample.

ICP-MS (Nexion, 2000, Agilent, Palo Alto, USA) was performed under the following conditions: radio frequency power of 1,350 w, carrier gas flow rate of 0.94 L/min, auxiliary gas flow rate of 0.40 L/min, helium flow rate of 4.5 mL/min, atomization chamber temperature of 2 °C, sample lifting rate of 0.3 r/s, sampling depth of 7 mm, resident time of 50 ms, nebulizer of PFA. The sampling cone was cone nickel, the collection mode was set to peak-hopping, and the number of scans was six times.

#### 2.3.2 Quality control

Blank control and standard samples were digested according to the above digestion method, and a quantitative standard curve was established, using Sc, Ge, In, Rh, Re, and Bi as internal standards. The limit of detection (LOD) was defined as the concentration corresponding to three times the standard deviation of the blank sample. For the elemental content of *C. equisetifolia* roots and leaves, the plant tissue standard (GBW07602) was used for comparison, and the soil elemental content was compared with the soil standard (GBW07403). The same digestion steps and determination methods were applied to the standards and samples. Each sample was determined in three independent replicates.

### 2.4 Statistical analysis

Excel 2017 software was used to categorize raw data and calculate means and variances. SPSS Statistics 21.0 software was used for *T*-tests and correlation analysis. RStudio software (R version 4.2.3) was used to produce heat maps (R library: pheatmap 1.0.12), box plots (R library: gghalves 0.1.4), principal component plots (R library: ggbiplot 0.55), volcano plots (R library: ggplot2 3.4.0), redundancy analysis (R library: vegan 2.6.4), and correlation matrix analysis (R library: linkET 0.0.7.1) [21]. The TOPSIS entropy weight statistical analysis was conducted on the SPSSAU online platform.

### 2.5 Ethical statement and permits

*Casuarina equisetifolia* is not an endangered or protected species. No specific institutional, national, or international permits were required for the collection of fresh roots and leaves used in this study. All sampling procedures were conducted in accordance with local regulations and standard field practices.

**Ethical approval:** The conducted research is not related to either human or animal use.

## 3 Results and discussion

### 3.1 Validation of quality control methods

The quality analysis of the plant tissue standard (GBW07602) showed (Table S1) that 46 elements were detected, and the recoveries of each element ranged from 86.21 % to 107.31 %. The quality analysis of the soil standard (GBW07403) showed (Table S2) that 64 elements were detected, and the recoveries of each element ranged from 85.15 % to 108.51 %. These results indicate that the extraction and detection methods used in the experiment are feasible, and the test results can be used for further analysis.

### 3.2 Effect of continuous planting on the elemental content of rhizosphere soil, roots, and leaves of *C. equisetifolia*

**Figure 1:**
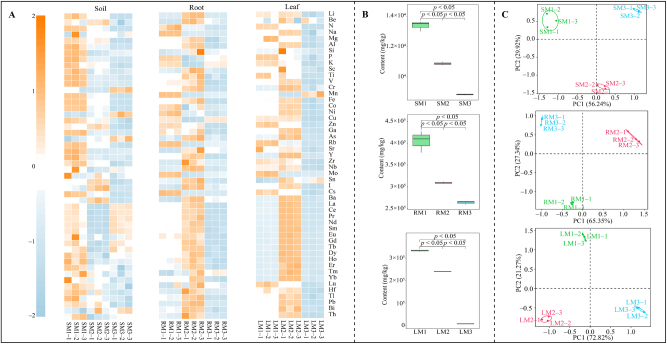
Effect of continuous planting on elemental content of rhizosphere soil, root and leaf of *C. equisetifolia*. M1: First planting; M2: Second continuous planting; M3: Third continuous planting; S: soil; R: Root; L: Leaf; (A) heat map of changes in elemental content in rhizosphere soil, roots and leaves of *C. equisetifolia* with different numbers of continuous plantings; (B) analysis of total elemental content in rhizosphere soil, roots and leaves of *C. equisetifolia*with different numbers of continuous planting; (C) principal component analysis of elemental content in rhizosphere soil, roots and leaves of *C. equisetifolia*with different numbers of continuous planting.

Many elements are involved in plant growth and development, helping plants cope with environmental changes, improve growth, and increase plant yield and quality [22]. However, soils have limited elemental content, and long-term agricultural activities can alter the content and distribution of elements in the soil, which in turn affects plant growth, especially when the same plant is grown in continuous monoculture on the same plot [23]. In this study, we analyzed the effect of continuous planting on the elemental content of rhizosphere soil (S), roots (R), and leaves (L) of *C. equisetifolia* and found (Figure 1A) that a total of 50 elements were detected in rhizosphere soil, roots, and leaves of *C. equisetifolia*. Further analysis of the total elemental content revealed (Figure 1B) that the total elemental content of the rhizosphere soil, roots, and leaves of *C. equisetifolia* showed a significant decreasing trend with an increase in the number of continuous plantings (M1–M3), as shown by SM1 (13.38 g/kg) > SM2 (10.89 g/kg) > SM3 (8.86 g/kg), RM1 (405.27 g/kg) > RM2 (309.43 g/kg) > RM3 (264.09 g/kg), and LM1 (333.07 g/kg) > LM2 (240.44 g/kg) > LM3 (7.02 g/kg). The results of principal component analysis of element content showed (Figure 1C) that the elemental content of the rhizosphere soil, roots, and leaves of *C. equisetifolia* could be effectively differentiated by the number of continuous plantings, with the overall contribution of the principal component to differentiate the element content of the rhizosphere soil being 86.1 %, that of the principal component to differentiate the element content of the roots being 92.6 %, and that of the principal component to differentiate the element content of the leaves being 94.1 %. These results indicate that there were significant differences in the element content in the rhizosphere soil, roots, and leaves of *C. equisetifolia* depending on the number of continuous plantings. In the process of plant growth, in addition to macro and trace elements, other mineral elements also play an important role. However, continuous planting of the same plant on the same plot for a long period of time is highly likely to cause significant changes in the content of different elements in the soil, which in turn effects the uptake and utilization of these elements by the plant [24], 25]. It can be seen that continuous planting is highly likely to lead to a decrease in the total content of elements in the rhizosphere soil of *C. equisetifolia*, and a decrease in the ability of roots and leaves to absorb these elements, which in turn may affect the growth of *C. equisetifolia*.

### 3.3 Effect of continuous planting on element enrichment of *C. equisetifolia* roots and leaves

**Figure 2:**
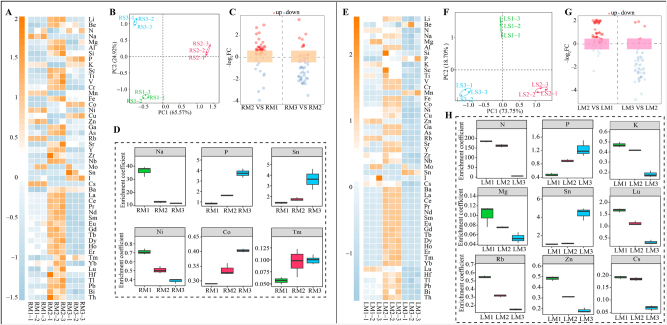
Effect of continuous planting on elemental enrichment coefficients in root and leaf of *C. equisetifolia*. M1: First planting; M2: Second continuous planting; M3: Third continuous planting; R: Root; L: Leaf; (A) heat map of elemental enrichment coefficients of *C. equisetifolia* roots with different numbers of continuous planting; (B) PCA analysis of elemental enrichment coefficients of *C. equisetifolia* roots with different numbers of continuous planting; (C) volcano plot screening for elements with differential enrichment coefficients in *C. equisetifolia* roots with different numbers of continuous planting; (D) main elements with changes in the enrichment coefficient of *C. equisetifolia* roots resulted by continuous planting; (E) heat map of elemental enrichment coefficients of *C. equisetifolia* leaves with different numbers of continuous planting; (F) PCA analysis of elemental enrichment coefficients of *C. equisetifolia* leaves with different numbers of continuous planting; (G) volcano plot screening for elements with differential enrichment coefficients in *C. equisetifolia* leaves with different numbers of continuous planting; (H) main elements with changes in the enrichment coefficient of *C. equisetifolia* leaves resulted by continuous planting.

Plant enrichment capacity for soil elements is usually assessed by enrichment coefficients, which reflect plant uptake and accumulation capacity for soil elements [26]. Therefore, based on the above study, elemental enrichment coefficients in the roots and leaves of *C. equisetifolia* were further converted and analyzed in this study. The results showed that the elemental enrichment coefficients in the root system of *C. equisetifolia* changed significantly with an increase in the number of continuous plantings (Figure 2A). The results of PCA analysis (Figure 2B) showed that the enrichment coefficients of elements in the roots of *C. equisetifolia* could be effectively differentiated by different numbers of continuous plantings, with the overall contribution of the principal components reaching 90.5 %. It can be seen that continuous planting had a significant effect on the uptake of different elements in *C. equisetifolia* roots. Accordingly, the volcano plot was further used in this study to screen for elements with differences in enrichment coefficients, and the results showed (Figure 2C and D) that the enrichment coefficients of Na and Ni in *C. equisetifolia* roots tended to decrease with an increase in the number of continuous plantings, while the enrichment coefficients of P, Sn, Co, and Tm tended to increase. Na has been reported to be important for water balance, photosynthesis, cell wall synthesis and maintenance, and cell membrane stability in plants [27]. Ni is extremely important in plant nitrogen metabolism and is a component of the urease, and Ni deficiency severely inhibits nitrogen conversion [28]. P and Co promote plant root growth [29], 30], but Sn and Tm have significant inhibitory effects on plant root growth [31], 32]. It can be seen that continuous planting leads to a decrease in the Na and Ni enrichment capacity of *C. equisetifolia* root, which in turn may hinder root cell division and nitrogen uptake. Moreover, as the number of continuous plantings increased, *C. equisetifolia* root showed increased enrichment of P and Co for root growth, but enrichment of Sn and Tm, which are inhibitory to root growth, was also increased at the same time. It can be concluded that continuous planting significantly affects the uptake of elements by *C. equisetifolia* root, which may in turn affect root growth.

Further analysis of leaf element enrichment showed that the elemental enrichment coefficients of *C. equisetifolia* leaves changed significantly as the number of continuous plantings increased (Figure 2E). The results of PCA analysis (Figure 2F) showed that enrichment coefficients of *C. equisetifolia* leaf elements could be effectively differentiated by different numbers of continuous plantings, with the overall contribution of principal components reaching 92.5 %. It can be seen that continuous planting also resulted in significant effects on the uptake of different elements by *C. equisetifolia* leaves. A volcano plot was further used to screen for elements with differences in enrichment coefficients, and the results showed (Figure 2G and H) that as the number of continuous plantings increased, enrichment coefficients of N, K, Mg, Lu, Rb, Zn, and Cs tended to decrease in *C. equisetifolia* leaves, while enrichment coefficients of P and Sn tended to increase. N, K, Mg, and Zn are all reported to be important elements for chlorophyll synthesis, photosynthesis, and carbon metabolism in plants and are extremely important for plant growth [33], [34], [35], [36]. Lu and Rb are beneficial for promoting plant growth, where Rb is also beneficial for promoting K and Na uptake in plants [37], 38]. Cs is toxic to plants, and plants selectively take up large amounts of Cs when they suffer from K deficiency [39]. Plants under environmental stress increase the uptake and translocation of P from the root system, increase P content in plant leaves, and promote flowering and early maturity [40]. Sn, as explained earlier, is toxic to plant growth. It can be seen that continuous planting of *C. equisetifolia* reduced the ability of its leaves to enrich elements such as N, K, Mg, Lu, Rb, and Zn, which may in turn reduce photosynthetic capacity. At the same time, continuous planting leads to the creation of a continuous planting obstacle in *C. equisetifolia*, followed by an increased P-enrichment capacity of the leaves, which may promote flowering, early maturity, and premature senescence.

The above analysis indicates that continuous planting tends to lead to a decrease in the enrichment capacity of beneficial elements (Na, Ni) related to root growth in *C. equisetifolia* roots, and a decrease in the enrichment capacity of beneficial elements (N, K, Mg, Lu, Rb, and Zn) related to photosynthesis, carbon metabolism, and nitrogen metabolism in leaves, while increasing the accumulation of harmful elements Sn and Tm, and the accumulation of P, which leads to premature senescence of *C. equisetifolia*. Continuous planting hinders the uptake of elements by *C. equisetifolia* and affects its growth.

### 3.4 Effect of continuous planting on elemental uptake and transport of *C. equisetifolia*

**Figure 3:**
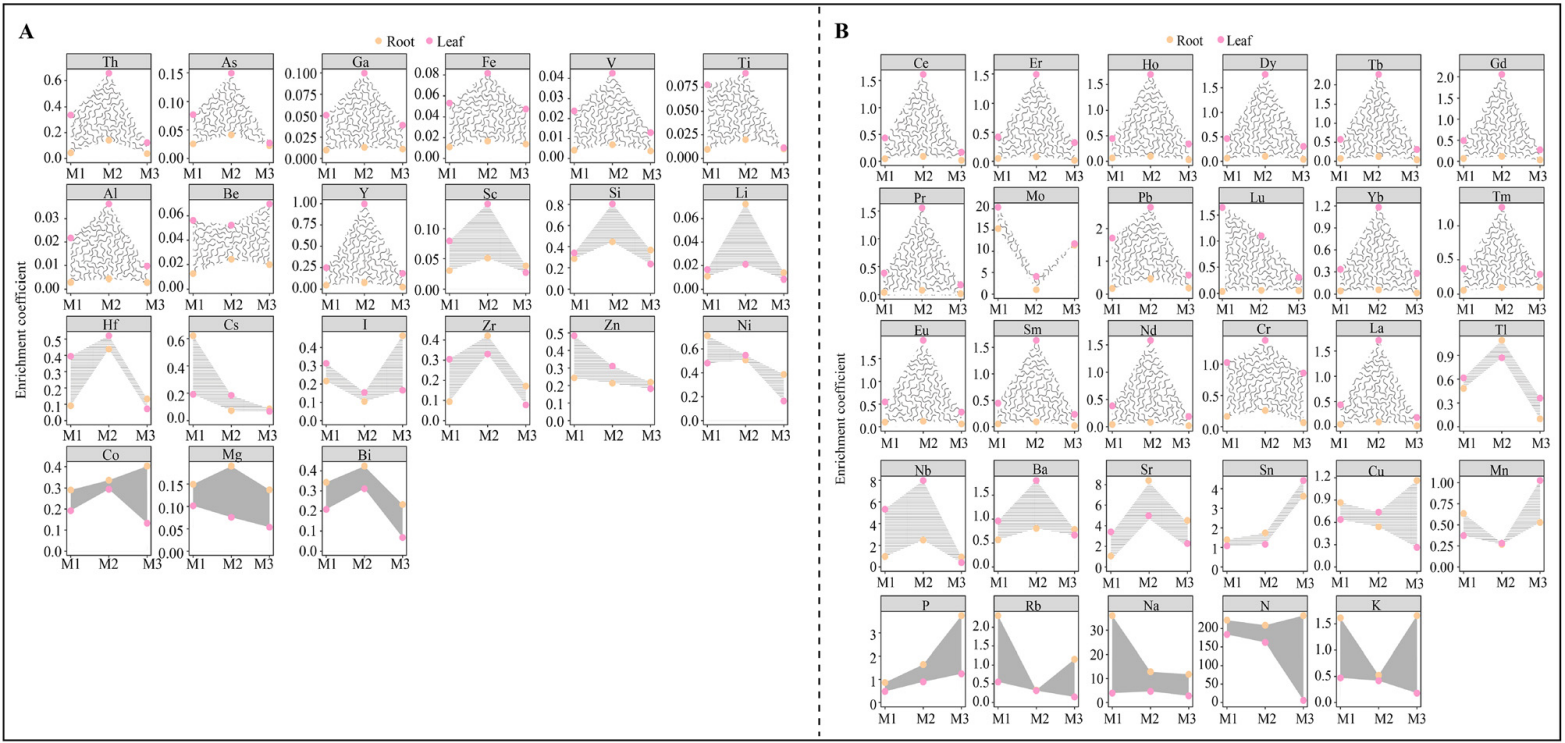
Analysis of differences in elemental enrichment coefficients in roots and leaves of *C. equisetifolia*with different numbers of continuous planting. M1: First planting; M2: Second continuous planting; M3: Third continuous planting; (A) elements with an enrichment coefficient of less than 1 in both roots and leaves of *C. equisetifolia* with different numbers of continuous plantings; (B) elements with an enrichment coefficient of more than 1 in roots or leaves of *C. equisetifolia* with different numbers of continuous planting.

In order to further analyze the effect of continuous planting on the uptake and transport of elements in *C. equisetifolia*, elemental enrichment coefficients in roots and leaves of *C. equisetifolia* with different numbers of continuous plantings were jointly analyzed in this study. The results showed (Figure 3) that of the 50 elements in the roots and leaves of *C. equisetifolia* with different numbers of continuous plantings, 21 elements had enrichment coefficients of less than 1 in both roots and leaves (Figure 3A), while 29 elements had enrichment coefficients greater than 1 in either roots or leaves (Figure 3B). The enrichment coefficient is an important indice for evaluating the plant’s ability to accumulate a specific element. When the enrichment coefficient is greater than 1, it indicates that the plant has a strong ability to accumulate this element, and vice versa [41]. Accordingly, the 29 elements with enrichment coefficients greater than 1 in the roots or leaves of *C. equisetifolia* were used for further analysis in this study.

**Figure 4:**
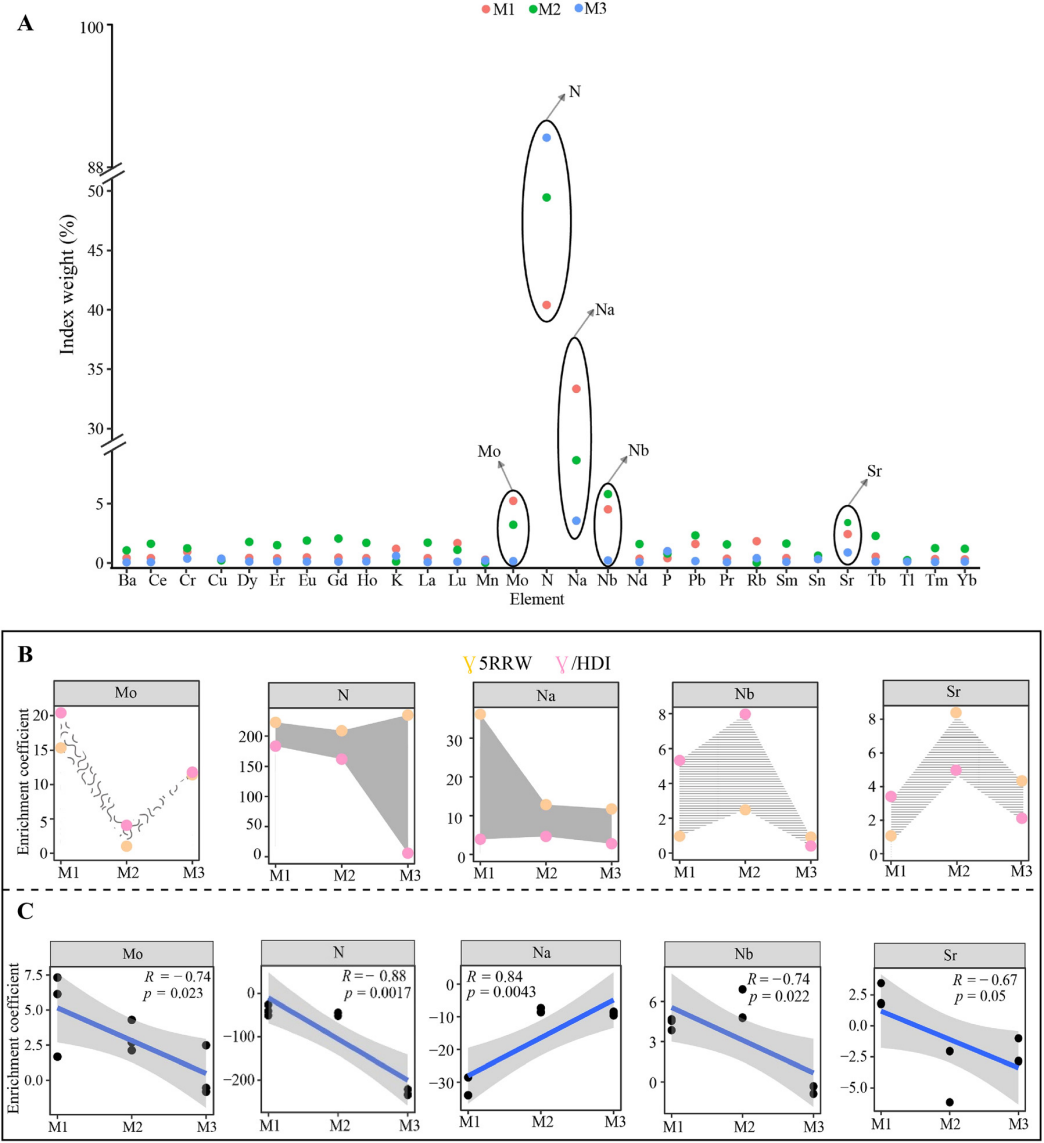
Effect of continuous planting on element uptake and transport in roots and leaves of *C. equisetifolia*. M1: First planting; M2: Second continuous planting; M3: Third continuous planting; (A) weighting analysis of the effect of continuous planting on the enrichment coefficient of different elements in *C. equisetifolia*roots and leaves; (B) effect of different numbers of continuous planting on the enrichment coefficient of key elements in *C. equisetifolia*roots and leaves; (C) effect of continuous planting on the transport of key elements from *C. equisetifolia* roots to leaves.

TOPSIS is a ranking method for approximating ideal solutions, commonly used in multi-indice decision evaluation. It ranks elements by detecting the integrated distance between the evaluation indices and the target, thus determining the degree of influence of the evaluation indices on the target [42]. Accordingly, TOPSIS was used in this study to analyze the effect of continuous planting of *C. equisetifolia* on the enrichment coefficients of 29 elements in roots and leaves. The results showed (Figure 4A) that the top five elements most affected by continuous planting in both roots and leaves of *C. equisetifolia* were N, Na, Nb, Mo, and Sr, with influence weights ranging from 76.21 % to 99.91 %, 3.92 %–62.88 %, 0.23 %–11.31 %, 0.16 %–9.86 %, and 0.97 %–4.58 %, respectively. Further analysis revealed that with the increase in the number of continuous plantings, the enrichment coefficients of *C. equisetifolia* roots showed a decreasing and then increasing trend for Mo and N, an increasing and then decreasing trend for Nb and Sr, and a steady decreasing trend for Na (Figure 4B). Secondly, with the increase in the number of continuous plantings, the enrichment coefficients of *C. equisetifolia* leaves showed a decreasing and then increasing trend for Mo, a steady decreasing trend for N, and an increasing and then decreasing trend for Na, Nb, and Sr (Figure 4B). The difference in enrichment coefficients between leaves and roots was used to simulate a trend line to analyze the ability of elements to be transported from roots to leaves of *C. equisetifolia*. The results showed (Figure 4C) that as the number of continuous plantings increased, *C. equisetifolia*’s ability to transport Mo, N, Nb, and Sr from roots to leaves decreased significantly, while the ability to transport Na increased. Although the ability to transport Na increased, its enrichment capability in both roots and leaves decreased significantly, and its content continued to decline. Mo and *N* have been reported to play important roles in plant chlorophyll synthesis and photosynthesis, which promote plant growth [33], 43]. Nb has growth-promoting activity in plants and enhances plant resistance to environmental stresses [44]. Sr promotes plant photosynthetic capacity, although high concentrations of Sr can stress plant growth [45]. As previously mentioned, Na is critical for water balance, photosynthesis, cell wall synthesis, maintenance, and cell membrane stability in plants [27]. It can be seen that continuous planting significantly reduced the ability of *C. equisetifolia* roots to absorb and transport elements, particularly Mo, N, Nb, and Sr, which in turn hindered its growth.

### 3.5 Effect of continuous planting on physiological indices of *C. equisetifolia* roots and leaves

**Figure 5:**
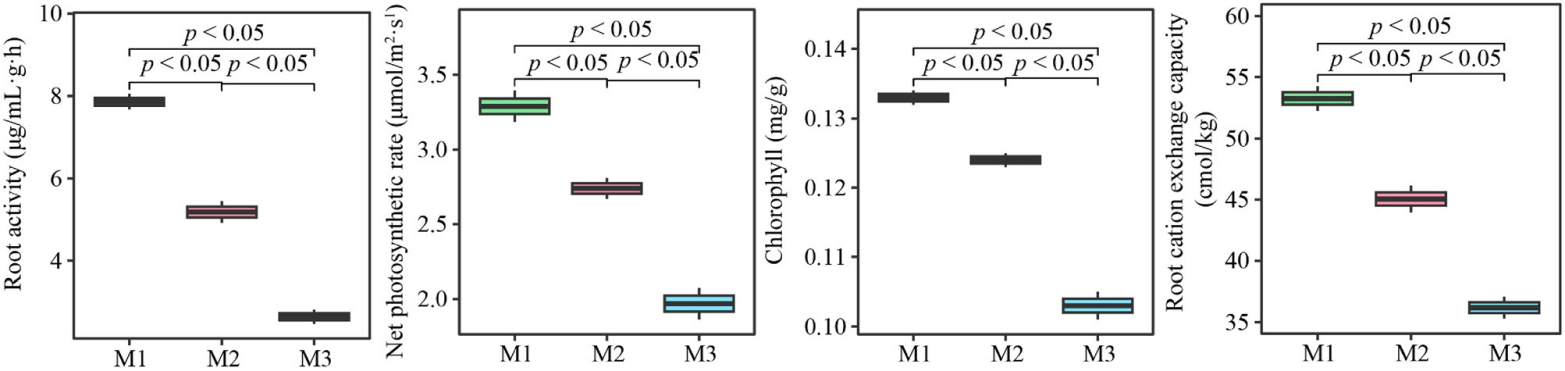
Effect of continuous planting on physiological indices of *C. equisetifolia* roots and leaves. M1: First planting; M2: Second continuous planting; M3: Third continuous planting.

The above analysis found that continuous planting led to differences in the uptake, enrichment, and transport of elements in the roots and leaves of *C. equisetifolia*. Functional analysis of the key differential elements indicated that continuous planting may lead to a reduction in the uptake capacity of elements in the roots, growth inhibition, and a decrease in the photosynthetic capacity of the leaves. It has been reported that root growth and its ability to absorb elements can be evaluated using root activity and root cation exchange capacity, while chlorophyll content and the net photosynthetic rate can be used to assess the photosynthetic capacity of plants [45], [46], [47]. Accordingly, this study further measured the root activity, root cation exchange capacity, chlorophyll content, and net photosynthetic rate of *C. equisetifolia* under different numbers of continuous plantings. The results showed (Figure 5) that with the increase in the number of continuous plantings (M1-M3), the root activity of *C. equisetifolia* decreased from 7.87 to 2.64 μg/mL g h, root cation exchange capacity decreased from 53.26 to 34.19 cmol/kg, chlorophyll content of leaves decreased from 0.13 to 0.10 mg/g, and the net photosynthetic rate decreased from 3.29 to 1.97 μmol/m^2^·s. These results clearly demonstrate that continuous planting leads to stunted root growth and reduced photosynthetic capacity of *C. equisetifolia*.

### 3.6 Interaction analysis of enrichment coefficients of key elements and physiological indices in roots and leaves of *C. equisetifolia*

**Figure 6:**
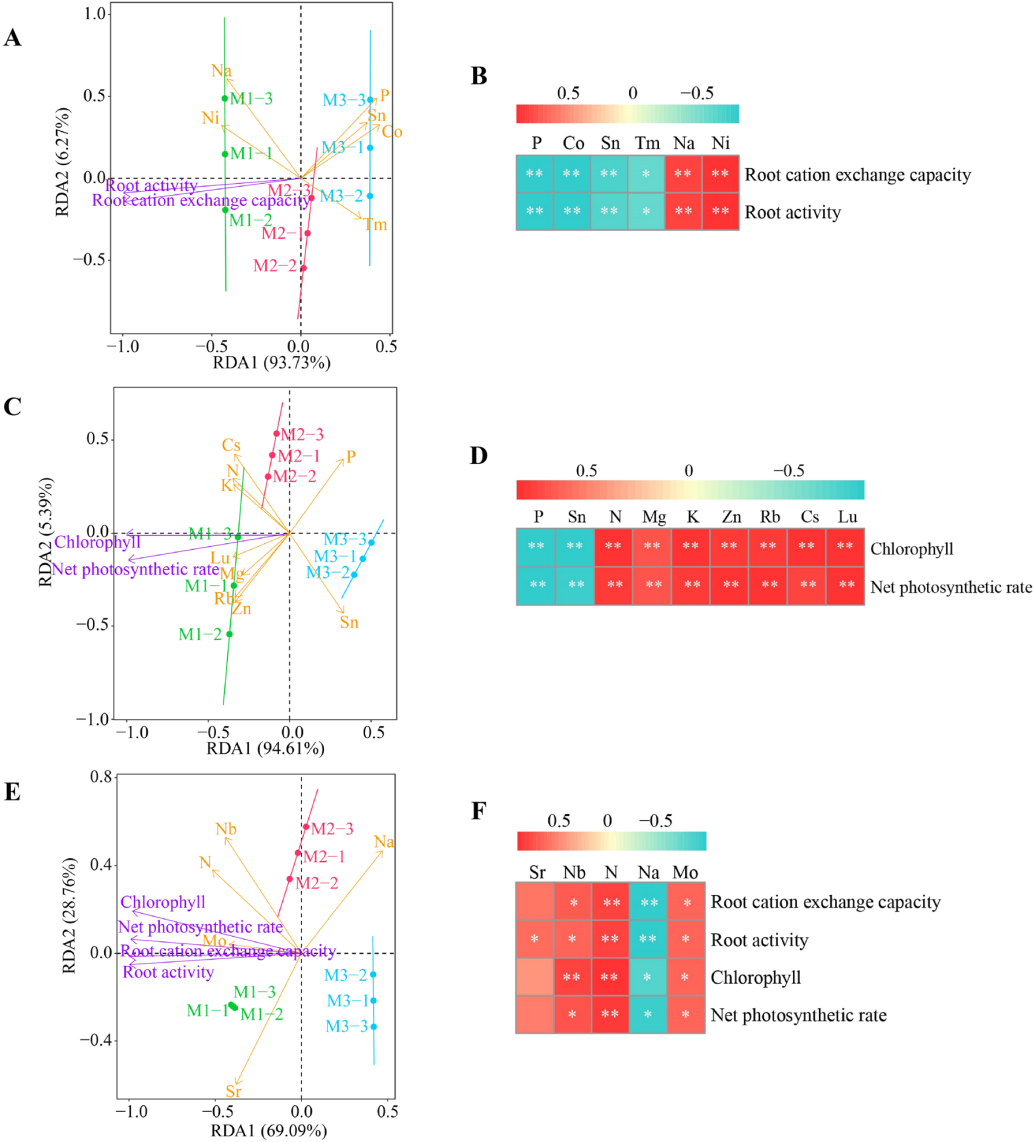
Interaction analysis of enrichment coefficients of key elements and physiological indinces of *C. equisetifolia* roots and leaves. M1: First planting; M2: Second continuous planting; M3: Third continuous planting; (A) redundancy analysis of enrichment coefficients of key elements in *C. equisetifolia* roots with physiological indices; (B) correlation analysis of enrichment coefficients of key element of *C. equisetifolia* roots with physiological indices; (C) redundancy analysis of enrichment coefficients of key elements in *C. equisetifolia* leaves with physiological indices; (D) correlation analysis of enrichment coefficients of key element of *C. equisetifolia* leaves with physiological indices; (E) redundancy analysis of enrichment coefficients of key elements with physiological indices during *C. equisetifolia* root-to-leaf transport. (F) Correlation analysis of enrichment coefficients of key elements with physiological indices during root-to-leaf transport of *C. equisetifolia*.

Based on previous studies, this study further analyzed the interaction between key element enrichment coefficients and physiological indices of *C. equisetifolia* roots and leaves. The results of redundancy analysis of the enrichment coefficients of key elements in the roots of *C. equisetifolia* with physiological indices showed (Figure 6A) that the main indices associated with M1 were Na, Ni, root activity, and root cation exchange capacity, whereas the main indices associated with M3 were P, Sn, Co, and Tm. The results of correlation analysis (Figure 6B) showed that root activity and root cation exchange capacity were significantly and positively correlated with Na and Ni, while they were significantly and negatively correlated with P, Sn, Co, and Tm. The redundancy analysis of key element enrichment coefficients and physiological indices in *C. equisetifolia* leaves showed (Figure 6C) that M1-related indices were predominantly Cs, N, K, Lu, Mg, Rb, Zn, chlorophyll content, and net photosynthetic rate, while M3-related indeices were predominantly P and Sn. Correlation analysis (Figure 6D) showed that chlorophyll content and net photosynthetic rate were significantly positively correlated with Cs, N, K, Lu, Mg, Rb, and Zn, while they were significantly negatively correlated with P and Sn. Redundancy analysis of the enrichment coefficients of key elements during transport between the roots and leaves of *C. equisetifolia* showed (Figure 6E) that M1-related indices were mainly Nb, N, Mo, Sr, root activity, root cation exchange capacity, chlorophyll content, and net photosynthetic rate, while M3-related indices were mainly Na. Correlation analysis (Figure 6F) revealed that root activity, root cation exchange capacity, chlorophyll content, and net photosynthetic rate were significantly positively correlated with Nb, *N*, and Mo, and significantly negatively correlated with Na. In addition, root activity was significantly positively correlated with Sr. It can be seen that continuous planting of *C. equisetifolia* reduced the uptake and enrichment capacity of Na and Ni by roots, the enrichment capacity of N, K, Mg, Lu, Rb, and Zn in leaves, and the ability of Mo, N, Nb, and Sr to be transported from roots to leaves. This, in turn, led to a decrease in root activity, root cation exchange capacity, leaf chlorophyll content, and net photosynthetic rate of *C. equisetifolia*, which ultimately hindered its growth.

## 4 Conclusions

**Figure 7:**
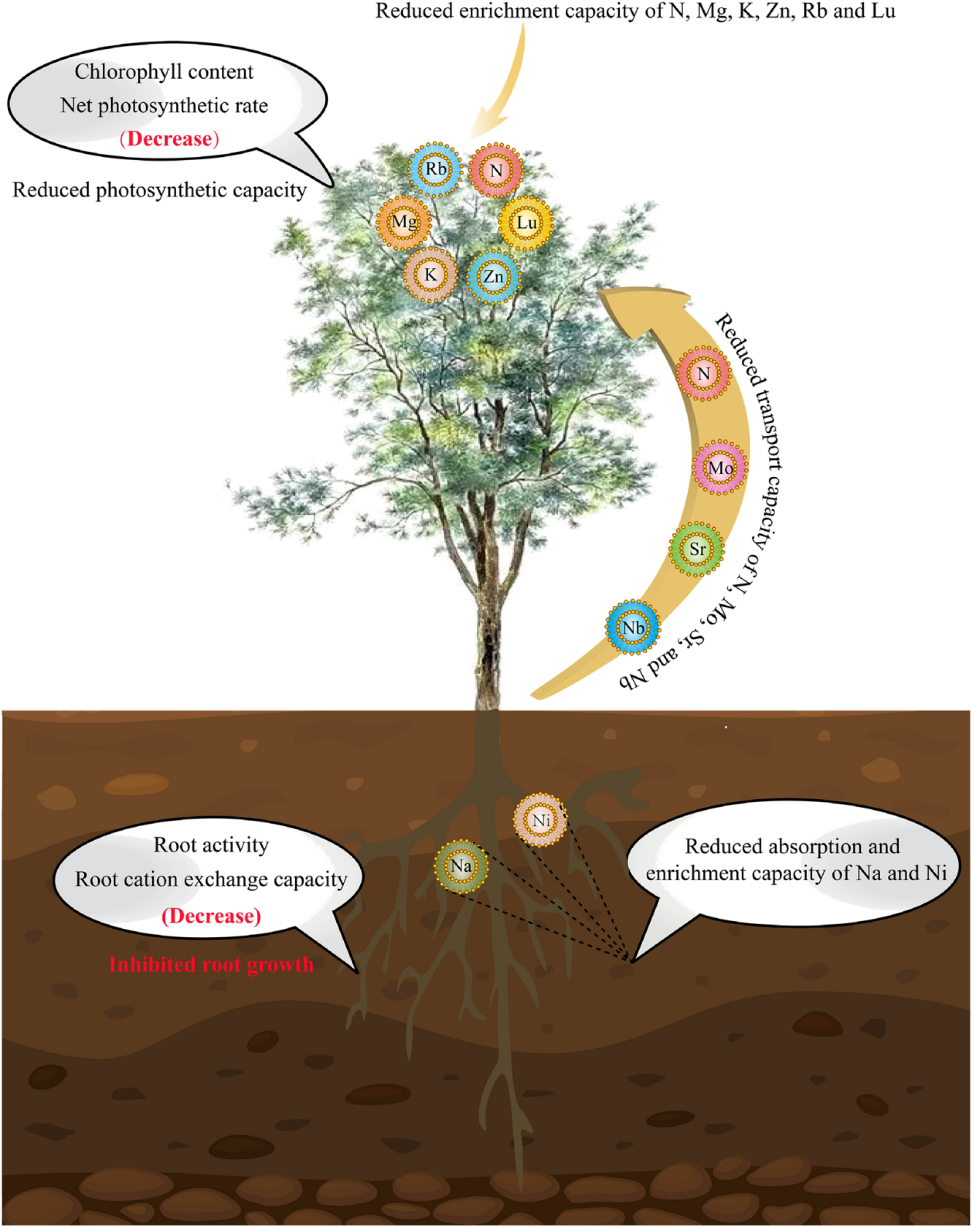
Mechanism of effect of continuous planting on elemental uptake and transport, physiological characteristics of *C. equisetifolia*.

In this study, we analyzed the effect of continuous planting on elemental uptake, transport, and physiological characteristics of *C. equisetifolia* roots and leaves. The results (Figure 7) showed that continuous planting reduced the elemental uptake and accumulation capacity of *C. equisetifolia* roots and leaves, which in turn hindered its growth. Specifically, continuous planting negatively affected the enrichment and transport of elements in the roots and leaves of *C. equisetifolia*. Key findings included reduced root uptake and enrichment capacity for Na and Ni, which subsequently led to reduced root activity and root cation exchange capacity. In additional, leaf enrichment capacity for N, K, Mg, Lu, Rb, and Zn was reduced, which in turn reduced leaf chlorophyll content and net photosynthetic rate. Continuous planting also led to a decline in root growth and photosynthetic capacity, while reducing the transport of Mo, N, Nb, and Sr from roots to leaves, further hindering *C. equisetifolia’s* growth. It is evident that continuous planting diminishes *C. equisetifolia*’s ability to absorb, enrich, and transport beneficial elements, limiting its growth. This study provides valuable insights for improving cultivation management practices and elemental regulation of *C. equisetifolia* in continuous planting systems. However, rhizosphere soil is a complex environment where microorganisms and plants interact. How continuous planting affects soil microorganisms, which in turn alters soil elemental composition and influences elemental uptake by *C. equisetifolia*, remains an area for further investigation.

## Supplementary Material

Supplementary Material
