# Supplementary material for: Continuous cropping changes the absorption and accumulation of elements in Casuarina equisetifolia, thus affecting its physiological properties
Source: Open Life Sci. 2026 Mar 2;21(1):20251283. doi: 10.1515/biol-2025-1283 (PMC12952211; doi:10.1515/biol-2025-1283)
Supplement: Supplementary file 1 — Supplementary Material [file j_biol-2025-1283_suppl_001.pdf]

Supplementary Materials

Table S1 Comparison of values of plant standard (GBW07602) determined by ICP-MS with certified values

Element	Certified value	Measured value	Recovery (%)	Element	Certified value	Measured value	Recovery (%)
Li	2.4±0.4	2.386±0.018	99.42±0.75	La	1.23±0.1	1.198±0.065	97.40±5.28
Be	0.056±0.014	0.053±0.005	94.64±8.93	Ce	2.4±0.3	2.287±0.029	95.29±1.21
B	34±7	34.189±1.289	100.56±3.79	Nd	1.1	1.023±0.048	93.00±4.36
Na	0.11±0.10	0.107±0.008	97.27±7.27	Sm	0.19±0.01	0.184±0.013	96.84±6.84
Mg	0.029±0.018	0.025±0.002	86.21±6.90	Eu	0.037±0.02	0.033±0.004	89.19±10.81
Si	0.058±0.04	0.061±0.005	105.17±8.62	Tb	0.026	0.023±0.002	88.46±7.69
N	0.012±0.02	0.011±0.001	91.67±8.33	Yb	0.063±0.014	0.059±0.004	93.65±6.35
P	830±40	847±21.256	102.05±2.56	Hf	0.14±0.02	0.124±0.009	88.57±6.43
K	0.085±0.05	0.081±0.004	95.29±4.71	W	0.06	0.052±0.006	86.67±10.00
Sc	0.31±0.03	0.319±0.013	102.90±4.19	Pb	7.1±1.1	6.975±0.176	98.24±2.48
Ti	95±18	94.372±3.289	99.34±3.46	Bi	0.22	0.198±0.013	90.00±5.91
V	2.4±0.3	2.308±0.167	96.17±6.96	Th	0.37±0.02	0.352±0.012	95.14±3.24
Cr	2.3±0.3	2.314±0.042	100.61±1.83	U	0.11	0.101±0.009	91.82±8.18
Mn	58±6	59.287±1.875	102.22±3.23	Al	0.214±0.022	0.198±0.014	92.52±6.54
Fe	1020±67	1094.582±35.468	107.31±3.48	S	0.032±0.03	0.031±0.003	96.88±9.38
Co	0.39±0.05	0.413±0.017	105.90±4.36	Ca	2.22±0.13	2.015±0.145	90.77±6.53
Ni	1.7±0.4	1.684±0.105	99.06±6.18	Sb	0.078±0.02	0.073±0.006	93.59±7.69
Cu	5.2±0.5	5.018±0.236	96.50±4.54	Nb	—	—	—
Zn	20.6±2.2	19.235±1.258	93.37±6.11	Sn	—	—	—
As	0.95±0.12	0.936±0.024	98.53±2.53	I	—	—	—
Br	2.4±0.04	2.307±0.093	96.13±3.88	Pr	—	—	—
Rb	4.2±0.2	4.089±0.265	97.36±6.31	Gd	—	—	—
Sr	345±11	353.721±8.286	102.53±2.40	Dy	—	—	—
Y	0.63	0.618±0.041	98.10±6.51	Ho	—	—	—
Mo	0.26±0.04	0.245±0.014	94.23±5.38	Er	—	—	—
Ag	0.027±0.006	0.024±0.002	88.89±7.41	Tm	—	—	—
Cd	0.14±0.06	0.129±0.013	92.14±9.29	Lu	—	—	—
Cs	0.27±0.03	0.257±0.024	95.19±8.89	Tl	—	—	—
Ba	19±3	18.045±0.983	94.97±5.17				

Note: Means ± standard error (SE) from three replications for each sample is shown. For GBW07602, the units of Na, Mg, Si, N, K, and S are mg/g; and those of other elements are μg/g, respectively.

Table S2 Comparison of values of soil standard (GBW07403) determined by ICP-MS with certified value

Element	Certified value	Measured value	Recovery (%)	Element	Certified value	Measured value	Recovery (%)
Li	55±3.7	52.243±1.187	94.99±2.16	Sn	2.5±0.4	2.475±0.153	99.00±6.12
Be	1.4±0.3	1.265±0.076	90.36±5.43	Te	0.04±0.15	0.038±0.001	95.00±2.50
B	23±4	22.184±1.384	96.45±6.02	I	9.4±1.2	9.106±0.364	96.87±3.87
C	0.035	0.037±0.001	105.71±2.86	Cs	3.2±6	3.134±0.178	97.94±5.56
Na	2.01±0.08	1.942±0.114	96.62±5.67	Ba	0.121±0.011	0.128±0.005	105.79±4.13
Mg	0.348±0.03	0.336±0.006	96.55±1.72	La	53±6	54.682±2.069	103.17±3.90
Si	34.86±0.135	32.489±1.156	93.20±3.32	Ce	39±6	40.265±1.894	103.24±4.86
N	0.064±0.004	0.061±0.001	95.31±1.56	Pr	4.8±0.4	4.702±0.146	97.96±3.04
P	320±8	314.186±8.679	98.18±2.71	Nd	18.4±2.4	19.056±1.062	103.57±5.77
K	1.261±0.002	1.189±0.029	94.29±2.30	Sm	3.3±0.3	3.208±0.203	97.21±6.15
Sc	5±0.6	4.806±0.214	96.12±4.28	Eu	0.72±0.06	0.714±0.019	99.17±2.64
Ti	0.224±0.012	0.231±0.004	103.13±1.79	Gd	4.7±0.6	4.593±0.215	97.72±4.57
V	36±4	34.187±1.126	94.96±3.13	Tb	0.49±0.09	0.481±0.016	98.16±3.27
Cr	32±6	30.985±1.138	96.83±3.56	Dy	2.6±0.2	2.506±0.148	96.38±5.69
Mn	304±21	308.265±7.465	101.40±2.46	Ho	1.46±0.14	1.398±0.103	95.75±7.05
Fe	0.389±0.062	0.379±0.008	97.43±2.06	Er	1.5±0.3	1.432±0.036	95.47±2.40
Co	5.5±1	5.136±0.116	93.38±2.11	Tm	0.28±0.06	0.272±0.009	97.14±3.21
Ni	12±27	13.021±0.402	108.51±3.35	Yb	1.7±0.3	1.624±0.087	95.53±5.12
Cu	11.4±1.6	11.895±0.428	104.34±3.75	Lu	0.29±0.03	0.265±0.012	91.38±4.14
Zn	31±4	32.046±0.843	103.37±2.72	Hf	14±2	14.896±0.665	106.40±4.75
Ga	31±5	30.183±0.702	97.36±2.26	Ta	0.76±0.2	0.712±0.033	93.68±4.34
Ge	1.9±0.4	1.804±0.054	94.95±2.84	W	0.95±0.29	0.931±0.0041	98.00±4.32
As	4.4±09	4.106±0.298	93.32±6.77	Hg	0.590±0.08	0.562±0.032	95.25±5.42
Br	4.3±0.7	4.138±0.265	96.23±6.16	Pb	26±4	27.248±0.984	104.80±3.78
Rb	85±6	82.065±3.012	96.55±3.54	Bi	0.17±0.06	0.159±0.006	93.53±3.53
Sr	380±25	391.284±10.267	102.97±2.70	Th	6±0.7	5.284±0.158	88.07±2.63
Y	15±2	16.032±0.403	106.88±2.69	U	1.3±0.4	1.107±0.036	85.15±2.77
Zr	246±21	257.138±6.089	104.53±2.48	Al	6.48±0.07	6.124±0.267	94.51±4.12
Nb	9.3±2.3	9.046±0.541	97.27±5.82	S	120±20	123.285±5.894	102.74±4.91
Mo	0.3±0.13	0.291±0.008	97.00±2.67	Ca	0.907±0.043	0.886±0.062	97.68±6.84
Ag	0.091±0.011	0.094±0.002	103.30±2.20	Sb	0.45±0.15	0.446±0.004	99.11±0.89
Cd	0.059±0.022	0.062±0.003	105.08±5.08	Tl	—	—	—
In	0.12±0.03	0.128±0.002	106.67±1.67				

Note: Means ± standard error (SE) from three replications for each sample is shown. For GBW07403, the units of C, Na, Mg, Si, N, K, Ti, Fe, Ba, Al, and Ca are mg/g; and those of other elements are μg/g, respectively.
